# Artificial intelligence convolutional neural networks map giant kelp forests from satellite imagery

**DOI:** 10.1038/s41598-022-26439-w

**Published:** 2022-12-23

**Authors:** L. Marquez, E. Fragkopoulou, K. C. Cavanaugh, H. F. Houskeeper, J. Assis

**Affiliations:** 1 CCMAR - Center of Marine Sciences, University of the Algarve, 8005-139 Faro, Portugal; 2 Department of Geography, University of California, Los Angeles, CA USA

**Keywords:** Image processing, Ecological modelling

## Abstract

Climate change is producing shifts in the distribution and abundance of marine species. Such is the case of kelp forests, important marine ecosystem-structuring species whose distributional range limits have been shifting worldwide. Synthesizing long-term time series of kelp forest observations is therefore vital for understanding the drivers shaping ecosystem dynamics and for predicting responses to ongoing and future climate changes. Traditional methods of mapping kelp from satellite imagery are time-consuming and expensive, as they require high amount of human effort for image processing and algorithm optimization. Here we propose the use of mask region-based convolutional neural networks (Mask R-CNN) to automatically assimilate data from open-source satellite imagery (Landsat Thematic Mapper) and detect kelp forest canopy cover. The analyses focused on the giant kelp *Macrocystis pyrifera* along the shorelines of southern California and Baja California in the northeastern Pacific. Model hyper-parameterization was tuned through cross-validation procedures testing the effect of data augmentation, and different learning rates and anchor sizes. The optimal model detected kelp forests with high performance and low levels of overprediction (Jaccard’s index: 0.87 ± 0.07; Dice index: 0.93 ± 0.04; over prediction: 0.06) and allowed reconstructing a time series of 32 years in Baja California (Mexico), a region known for its high variability in kelp owing to El Niño events. The proposed framework based on Mask R-CNN now joins the list of cost-efficient tools for long-term marine ecological monitoring, facilitating well-informed biodiversity conservation, management and decision making.

## Introduction

Ongoing climate change is shifting the distribution of marine species worldwide^1,2^. Future climate projections suggest further range shifts, potentially driving major biodiversity losses^3–5^. Accordingly, the future maintenance of ecosystem functioning will likely depend on the regional persistence of structuring species^6^ such as giant kelp (Macrocystis pyrifera), the largest and most widespread kelp. This forest-forming species provides multiple ecosystem services, such as coastal protection, blue carbon sequestration, and nursery areas for numerous associated species, some of which have high economic value^7–9^.

Giant kelp forests are naturally resilient but recent changes reported in their distribution and abundance are undermining ecosystem services and unbalancing trophic interactions^5,10^. This has been particularly striking at equatorward distributional range limits, where poor nutrient conditions and high temperature anomalies^11–13^ have led to changes in populations worldwide^5^. Future projections for the species estimate further losses. For instance, even under low emission scenarios, giant kelp populations of Australia are projected to lose 79% of their potential suitable habitats, while under more aggressive scenarios, complete losses are expected^14^. As a result, systematic monitoring is required to track broadscale changes in kelp forests over time, and to discriminate the impacts of climate change from natural long-term variability^15,16^.

Remote monitoring of giant kelp is possible with satellite imagery due to the high near-infrared reflectance of dense floating canopies on the water surface^17^. Different sensors and classification techniques have been used to detect and reconstruct kelp coverage over time^17,18^. However, most of these techniques are based on spectral analyses of individual pixels (e.g., Multiple Endmember Spectral Mixture Analysis^19^), requiring high operational costs for image processing and algorithm optimization. The use of artificial intelligence semantic interpretation of satellite imagery, just like human perception extracts distinct features from images^20,21^, could advance the field, by enabling automatic detection of the easy to distinguish floating canopies of giant kelp forests^18^ with reduced costs.

Deep learning can automatically learn representations from images without human domain knowledge^22^. More specifically, convolutional neural networks (CNN) can distinguish features of different classes of objects from pre-annotated images and make accurate predictions^23^. Learning of CNNs can be boosted by data augmentation, in which the size of the training data set is artificially increased, and also by transfer learning, in which learning of the network begins with a prior knowledge^24,25^. This class of algorithms have been recently used in the marine context to identify, e.g., whales^25–27^, oyster reefs^28^ and features like ships, garbage patches and oil spills^27,29^, from satellite imagery with high performance. In the field of feature detection, the region-based CNN (R-CNN) algorithm was developed to extract the location of classified features, i.e., the parts or patterns of an object to be recognized^30^. This was improved in terms of detection speed (Faster R-CNN) by physical-like sampling mapping^31,32^ and regional reference networks sharing all convolution layers^32^. Later, the Mask R-CNN extended Faster R-CNN with a new branch (FCN) capable of predicting the features’ mask within the region recognition branch^33,34^. This algorithm achieves object outline detection with remarkable performance^32^, opening the possibility of detecting the coverage of giant kelp forests from satellite imagery (e.g., square meters of kelp forests in a given region).

In the present study, we propose the use of mask region-based convolutional neural networks (Mask R-CNN) to automatically assimilate data and detect giant kelp coverage from satellite imagery (Landsat Thematic Mapper). In addition, we demonstrate the ability of the method by reconstructing a time series of kelp coverage with 32 years of satellite data from a particular region of interest: Baja California, Mexico, where El Niño events have recurrently impacted the distribution of giant kelp forests^12,35^. The proposed method aims for automatic, regular, and updated monitoring of giant kelp forests, facilitating well-informed biodiversity conservation, management and decision making (e.g., marine protected areas). The outputs generated can be used in explanatory modelling for a better understanding of ongoing and projected ecosystem dynamics and services.

## Methods

We build a Mask R-CNN framework learning from satellite data and tuned with optimal hyperparameterization to generalize predictions of giant kelp coverage. This was used to reconstruct a long-term time series of kelp coverage in the species equatorward distributional range limits—Baja California (Mexico). Mask R-CNN is an excellent candidate method for giant kelp identification and segmentation because it successfully combines the high-performance algorithms of Faster R-CNN for target identification and FCN for mask prediction, boundary regression and classification^36^.

All analysis and experiments were performed in Python programming language (v3.7.1) with the frameworks of Matterport Inc.^37^, Keras (v2.0.8) and Tensorflow (v1.13.1), using a desktop computer with 40 Intel Xeon cores (hyperthreading technology) and 128 Gb of memory, and running Ubuntu 18.04. With these resources, the models took approx. 5 days to train. All code developed is permanently available at github.com/jorgeassis/maskRCNN.

### Model species

The giant kelp *Macrocystis pyrifera* is a coastal species that can be found from temperate to subpolar latitudes. In the northern hemisphere, it is distributed from Alaska to Baja California (Mexico), while in the southern hemisphere, from Peru to Argentina, as well as in Australia, South Africa, New Zealand and some sub-Antarctic Islands.

Giant kelp forms dense floating canopies on the ocean surface that are clearly perceived in satellite imagery. In particular, the reflectance signature of the canopies is mostly in the near-infrared making them easily distinguished from the surrounding waters, which absorb nearly all energy at this wavelength^19^. In addition, giant kelp is the dominant species with floating canopies in the study region^38^, greatly simplifying the estimation of its coverage.

### Satellite imagery

**Figure 1:**
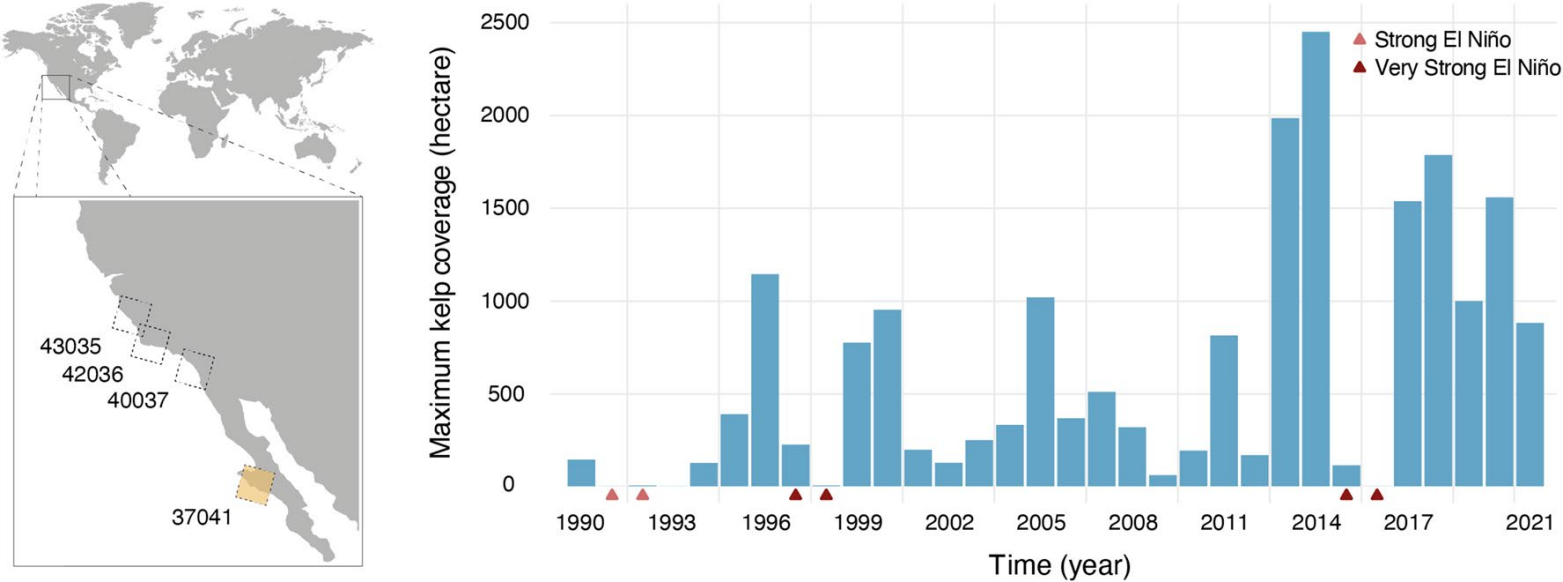
(Left panel) Regions where satellite imagery (Landsat Thematic Mapper) was obtained to develop Mask R-CNN models. Numbers refer to the scene code of Landsat. (Right panel) Maximum kelp coverage predicted for 32 years of satellite data of Baja California (Mexico; yellow square of the left panel), where El Niño events (red triangles) have recurrently impacted the equatorward distributional range limits of giant kelp. Figure generated in R computing language^60^ using the open-source landmass polygon provided by OpenStreetMap^61^.

**Figure 2:**
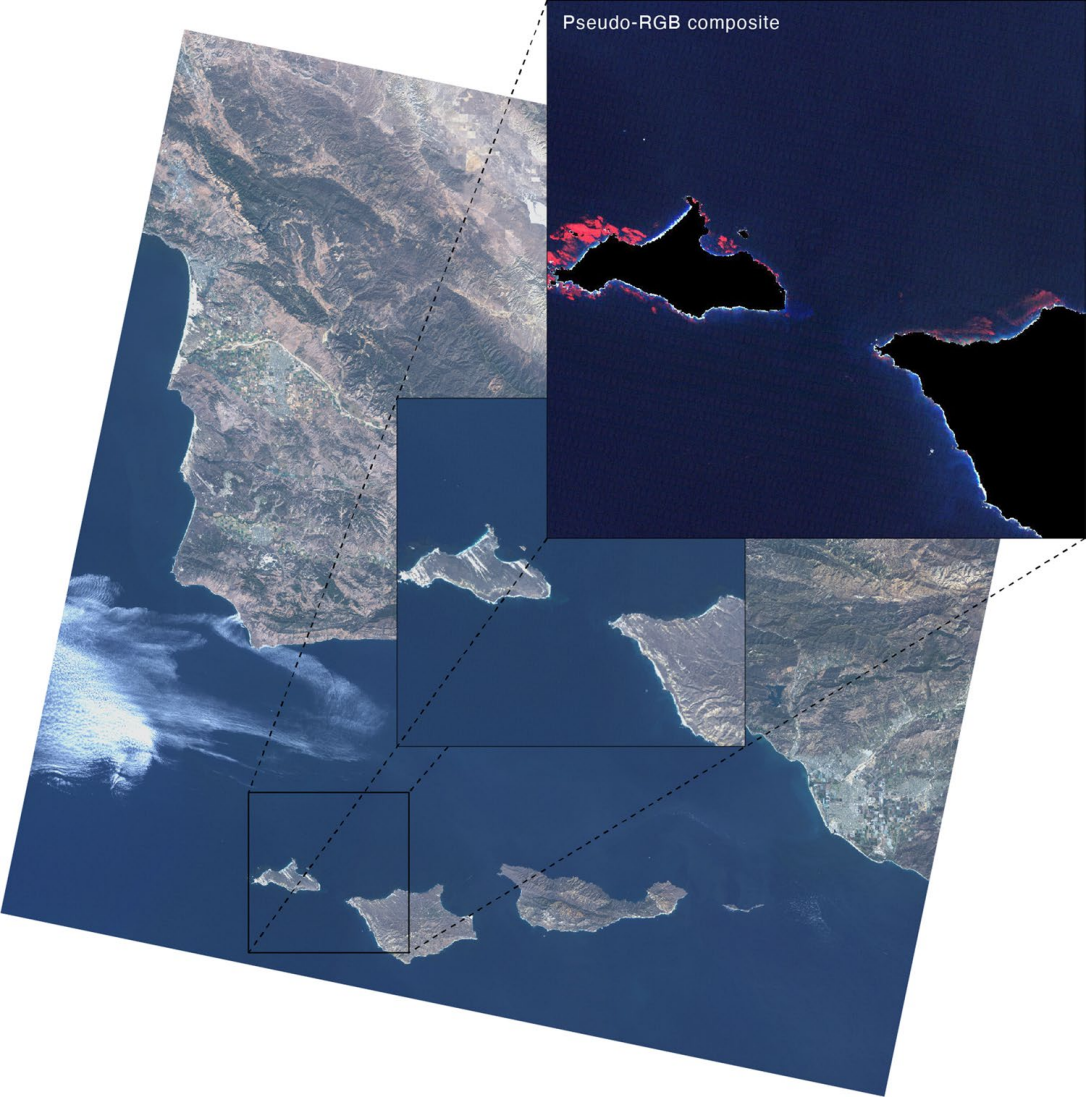
Example of a pseudo-RGB composite (with near-infrared, red and green bands), where floating canopies of giant kelp are easily perceived (depicted in red). Pseudo-RGB composites were produced from square tiles (1024 × 1024 pixels) of Landsat satellite images, which were preprocessed with a mask matching landmass (depicted in black). Figure generated in R computing language^60^ using an open-source Landsat satellite image, courtesy of the U.S. Geological Survey.

**Figure 3:**
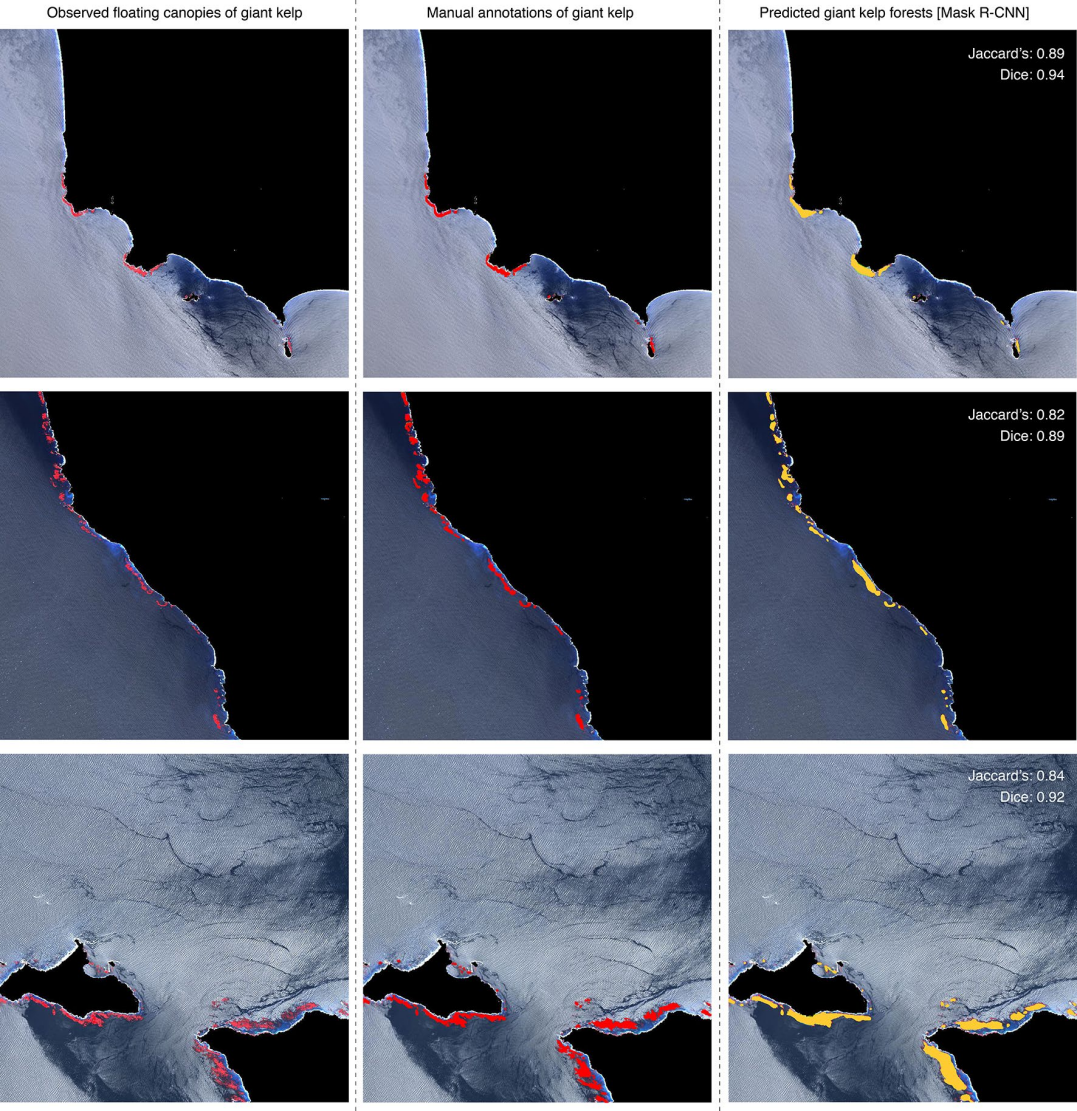
Example of 3 pseudo-RGB composites used in independent cross-validation. (Left panels) Observed floating canopies of giant kelp (depicted in red). (Central panels) Manual annotations of giant kelp made by experts (depicted in red). (Right panels) Predicted giant kelp forests with Mask R-CNN (depicted in yellow). Performance of predictions is shown with Jaccard’s index and Dice coefficient. An example of the outputs of Mask R-CNN including the bounding box detections of giant kelp are available in Supplementary Information (Figs. S9, S10). Figure generated in R computing language^60^ using an open-source Landsat satellite image, courtesy of the U.S. Geological Survey.

Satellite imagery was obtained from Landsat, a series of satellites with sensors acquiring multispectral imagery in 7 spectral bands at 30 m spatial resolution, with scenes covering an area of approx. 30,600 km^39^. Images were pooled from Google Earth Engine API^40^ for 3 scenes of the coast of California, USA, and one scene of Baja California, Mexico (Fig. 1), using the implemented atmospheric correction algorithm and the cloud cover filter adjusted to less than 5%. This retrieved a total of 130 images (USGS Landsat 5 and 8 Surface Reflectance Tier 1; Landsat 7 was not used due to known image artifacts)^41^ spanning from 1997 to 2021. Pseudo-RGB composites were generated by selecting the near-infrared (760 to 900 nm), the red and the green bands (Fig. 2), in line with recent studies published in the scope of remote sensing of kelp forests^15,42–44^. While the near-infrared band allows generating images of high contrast, considering the high reflectance of kelp canopies and the high absorption of water masses at this wavelength^19,45^, the additional bands (red and green) provide informative parameters to discriminate surface cover type and, for aquatic surfaces, particle content^46^. To avoid false positives associated with terrestrial detections of vegetation cover, landmasses were automatically masked using the^47^ dataset, as implemented in Google Earth Engine (Fig. 2). Due to the general small size of floating kelp canopies (Fig. 3), and to improve the computational process during model training, images were cropped into multiple tiles of 1024 × 1024 pixels (Fig. 2; 943,72 km^2^), therefore preserving the native resolution of satellite imagery^26^.

Tilled images with kelp were annotated by experts with VGG Image Annotator^48^ version 2.0 (www.robots.ox.ac.uk/~vgg/software/via/), a standalone software that stores information as JSON files. Kelp forests were manually digitized and labelled as “kelp”, a process that resulted in 3345 “kelp” polygons in 421 tiles.

### Model training

Considering the high variability in the spatial and temporal patterns of kelp forests^15,49^, as well as the dynamic of floating canopies in terms of contour and shape^50^, the image catalog was randomly split into 3 datasets: the training dataset with 75% of the catalog (317 tiles, containing 2368 “kelp” polygons totalizing 510.77 km^2^ of area), the testing dataset with 17.5% of the catalog (74 tiles, 537 “kelp” polygons, 192.89 km^2^ of area), and a final independent dataset to assess the performance of the model with 7.5% of catalog (30 tiles, 440 “kelp” polygons, 52.91 km^2^ of area). The average size of annotated “kelp” polygons was 0.33 km^2^ (± 0.51 km^2^ SD), in line with additional studies using Landsat to map kelp forests elsewhere^51^.

An experimental design based on the grid-search method was implemented to properly tune the optimal hyperparameterization of Mask R-CNN models (Table 1). This approach compared the performance of all combinations of hyperparameters in cross-validation^52,53^. In particular, different anchor sizes and learning rates were tested because they can significantly impact the performance of CNN models. Anchors are grids of squares with different sizes used to propose the location of objects, thus, choosing a proper size is essential to accurately detect giant kelp forests^54,55^. Learning rate controls how much the model changes each time its weights are updated, in response to the predicted error (each update is called an epoch^55^). A small learning rate may result in prolonged training, more prone to overfitting (i.e., complex fit describing random noise), while a large rate may result in a sub-optimal set of weights with reduced performance and generalization^26^. The effect of data augmentation was also tested in cross-validation. This technique artificially increases the volume of the training dataset by image transformation^56^. Images were randomly rotated by 90º steps, flipped from left to right, from top to down, and rescaled by 50% (i.e., a fourfold increase of the original data).

**Table 1:** Experiments performed with Mask R-CNN models to identify the best combination of hyperparameters (*DA* data augmentation, *LR* learning rate and *AS* anchor size) to predict giant kelp in Landsat satellite imagery.

#	Experiment conditions			Performance			Pairwise tests							
	DA	LR	AS	Jaccard I	Dice C	Overprediction	1	2	3	4	5	6	7	8
1	No	0.001/0.0001	32,64,128,256,512	0.753 ± 0.104	0.855 ± 0.072	0.194	–	*	*	*	*			*
2	**Yes**	**0.001/0.0001**	**32,64,128,256,512**	**0.874 ± 0.068**	**0.931 ± 0.039**	**0.064**	*	–			*	*	*	*
3	Yes	0.001/0.0001	16,32,64,128,256	0.853 ± 0.095	0.918 ± 0.060	0.103	*		–			*	*	
4	Yes	0.0001/0.00001	16,32,64,128,256	0.856 ± 0.088	0.920 ± 0.054	0.071	*			–	*	*	*	
5	Yes	0.0001/0.00001	32,64,128,256,512	0.824 ± 0.105	0.899 ± 0.073	0.094	*	*		*	–			
6	No	0.0001/0.00001	16,32,64,128,256	0.755 ± 0.150	0.851 ± 0.104	0.122		*	*	*		–		*
7	No	0.0001/0.00001	32,64,128,256,512	0.774 ± 0.145	0.865 ± 0.100	0.132		*	*	*			–	*
8	No	0.001/0.0001	16,32,64,128,256	0.822 ± 0.132	0.895 ± 0.091	0.119	*	*				*	*	–

The performance of each hyperparameter combination is shown as the average Jaccard’s index, Dice coefficient and overprediction of coverage (proportion of classification, ranging from 0 to 1). Pairwise tests were performed to identify the model with significantly different Jaccard index and Dice coefficient (asterisks depict significant p-values with Bonferroni correction). Bold highlight shows the optimal hyperparameter combination to detect giant kelp forests in the satellite imagery.

Models were trained with all combinations of the 3 hyperparameters in two steps: a step training the first 10 epochs for the head layers of the CNN, with classification and regression of the bounding boxes localizing giant kelp in the image; followed by training all layers in 50 epochs, a step which also trained the backbone of the model for edge detection. In the first 10 epochs of training the head layers, the learning rate was set as 10 times faster than when training all layers^57^. The models benefited from previous transfer learning consisting of starting the training process using the weights from a pre-trained model using the COCO dataset^25^, which contains 1.5 million object instances of 80 different categories^58^. During training, a loss function was generated to compare the performance of predictions through cross-validation against the testing dataset. The loss function of the implemented framework of Mask R-CNN is determined by the expression:

$$ {\text{Loss}}\;{\text{function}} = {\text{Classification}}\;{\text{Loss}} + {\text{Bounding}}\;{\text{Box}}\;{\text{Regression}}\;{\text{Loss}} + {\text{Mask}}\;{\text{Loss}}, $$

where the Classification Loss and Bounding Box Regression Loss are determined through cross-entropy as in the Faster R-CNN framework^31,32^, and reflect the ability of the model to classify kelp and to identify the regions of the image (i.e., bounding boxes) where kelp occurs. The Mask Loss is determined through binary cross-entropy per pixel^34^, for the images where kelp was classified, and reflects the ability of the model to identify the masks (i.e., the outlines) of kelp forests.

For each experiment, we choose the configuration of the epoch retrieving the minimal loss function and used it to evaluate the final accuracy of the model.

### Model evaluation and optimal parameterization

The models were evaluated against the independent dataset using the Jaccard index and the Dice coefficient, two methods based on the overlap between the predicted and annotated (observed) masks, i.e., regions with giant kelp. The Jaccard index penalizes inaccurate predictions in single instances, an approximate metric for worst-case performance, while the Dice coefficient is used as a general measurement of the model’s performance.

The Jaccard index (J) is defined as:

$$J= \frac{|A\cap B|}{|A\cup B|}=\frac{\left|\mathrm{A}\cap \mathrm{B}\right|}{\left|\mathrm{A}\right|+\left|\mathrm{B}\right|-|\mathrm{A}\cap \mathrm{B}|},$$

where *A* and *B* are the predicted and observed regions with giant kelp, respectively.

The Sørensen's Dice coefficient (DSC) is defined as:

$$DSC=\frac{2\left|A\cap B\right|}{\left|\mathrm{A}\right|+\left|B\right|},$$

where *A* and *B* are the predicted and observed regions with giant kelp, respectively.

To identify the optimal combination of hyperparameters, the Jaccard index and Dice coefficients were compared across all models. To this end, pairwise comparisons between experiments were performed using the non-parametric Mann–Whitney U test, which is equivalent to the two sample t-test for comparing the mean of two independent groups, but without the assumption of normality^59^. The model retrieving significantly higher Jaccard index and Dice coefficient was chosen as the optimal model configuration to detect giant kelp forests in the satellite imagery.

### Reconstruction of a giant kelp time series

To demonstrate the ability of Mask R-CNN to detect giant kelp coverage, the optimal model was used to reconstruct a time series of 32 years of data from Baja California in Mexico (Landsat path 37 and row 41, or scene 037041; 157 images with cloud cover less than 5% from 1990 to 2021; Fig. 1). Because satellite imagery is not consistently available per Landsat cycle, mostly due to different Landsat missions and high regional cloud coverage, the generated dataset was aggregated to the maximum coverage of kelp per year (average images per year: 18.25 ± 8.97). This way, the demonstration here proposed captures the inter-annual variability of giant kelp forests.

## Results

The optimal model configuration detecting giant kelp forests with higher performance was the one using data augmentation, a learning rate of 0.001 for the head layers and 0.0001 for the remaining layers, and anchor sizes set to 32, 64, 128, 256 and 512 (i.e., Experiment #2; Table 1). This resulted in an average Jaccard index and Dice coefficient of 0.874 ± 0.068 and 0.931 ± 0.039, respectively, and an average overprediction of kelp coverage of 0.064 (tested in the independent dataset). Pairwise tests comparing all hyperparameter combinations showed two additional models matching the performance of the previously described model (Experiments #3 and #4), both using data augmentation, but distinct learning rates and anchor sizes (Table 1). The overall losses assessed per experiment along the 50 epochs of training stages, as well as the epochs retrieving minimal losses, are available in Supplementary Information (Figs. S1–S8).

The optimal model was applied to 157 images, covering to 32 years of data (1990 to 2021) from Baja California Sur in Mexico. This time series showed high inter-annual variability, with high kelp coverage (> 5000 m^2^) in 1999, 2000 and 2005, and low (< 1000 m^2^) or no kelp coverage in 1991 to 1994, 1998, 2003, 2009 and 2016 (Fig. 1).

An example of giant kelp floating canopies manually annotated and predicted by the model is presented in Fig. 3.

## Discussion

This study proposes the use of mask region-based convolutional neural networks (Mask R-CNN) to detect giant kelp forests in satellite imagery. The implemented framework performed outline detection (i.e., coverage) with high performance and low levels of overprediction (Jaccard’s index: 0.87 ± 0.07; Dice index: 0.93 ± 0.04; over prediction: 0.06). A demonstration of the framework was performed with success by predicting to 32 years of satellite data of Baja California, Mexico. This reconstructed a time series of kelp coverage in a region known for its high variability in kelp forests owing to El Niño events^12,37^. The method now joins the list of cost-efficient and less time-consuming approaches for long-term marine ecological monitoring^28,62–64^.

The proposed application based on Mask R-CNN used the grid-search method to properly tune hyperparameterization. The performance of eight models fitting different hyperparameters was compared with independent data. Results showed higher performance in kelp detection when considering data augmentation, a learning rate of 0.001 for the head layers and 0.0001 for the remaining layers, and an anchor size of 32, 64, 128, 256, 512. The positive impact of data augmentation on the performance of CNN has been previously shown elsewhere^25,28^. This technique of virtually increasing training data can be particularly advantageous in reducing overfitting in small, highly structured datasets^65,66^, such as our case with kelp forests. Importantly, in the presence of data augmentation, the different anchor sizes and learning rates tested did not result in models with statistically different performances. These two hyperparameters only impacted the models not considering data augmentation, yet not in a straightforward way. The effect of each one was interdependent on the other, such that there was no pattern of performance and generalization change^26^ while reducing/increasing learning rate. The same for the different anchor scales tested, reflecting different grids of squares generating the region proposal network^67^. Accordingly, the grid-search method resulted in an appropriate approach to infer the best combination of such interdependent hyperparameters^5^.

The performance of our model tuned with optimal hyperparameters is comparable to additional marine applications using CNN to identify features in satellite or aerial imagery. Our results ranging between 0.87 and 0.93, depending on the index considered, are in line with the 0.94 reported for whale counting in Google Earth imagery^25^, the 0.85 for coral reefs identification in WorldView-2 and 0.80 in Planet satellite imagery^68^, the 0.89 to 0.97 for ships, garbage and oil spills recognition in Google Earth imagery^27^, and the 0.92 for shellfish reefs segmentation in high-resolution imagery from unmanned aircrafts^28^.

Despite the high performance achieved, the ability to detect kelp coverage was not flawless, and potential drawbacks of our framework should be acknowledged. The first is related to the resolution of the satellite imagery used (Landsat Thematic Mapper), which may be too coarse to allow proper detection of kelp forests, particularly when an area equivalent to a pixel is covered by less than 15%^69^. This means that CNN may find it difficult to differentiate sparse kelp forests from the background when pixels contain a mixture of land, water and kelp^69–71^. To overcome this, higher resolution imagery could be used with our Mask R-CNN framework, however, the available datasets are not completely open-source at decadal time scales like the Landsat Thematic Mapper. The second potential drawback has to do with the cloud detection algorithm used. Cloud contamination is a recurrent challenge in applications using satellite imagery^21,72^, and our study might not be the exception. Typically, clouds are identified and removed before data processing^73,74^. In our case, the effect of clouds overlapping kelp forests was dealt with by filtering images with a cloud cover of less than 5%, as implemented in Google Earth Engine API^40^. Yet, the potential presence of occasional clouds could have interfered in kelp detection, owing to changes in reflectance. Considering the automatic implementation proposed, it is not possible to measure such an effect, and only the future optimization of algorithms and sensors may overcome this^72^. The third potential drawback has to do with the high variability in the spatial patterns of kelp forests. Here, Mask R-CNN aimed to generalize the shape of kelp forests, but while some forests can be very dense and well-defined, others may not, making edges blurry and challenging the backbone, i.e., the modelling structure responsible for edge detection^75^. This might be the major reason behind kelp coverage being slightly overestimated, and behind the higher role of data augmentation, as virtually increasing the training data leads to a better generalization of features and increased robustness^65,66^.

To demonstrate the ability of our Mask R-CNN implementation to detect giant kelp coverage, we fed the model with 32 years of satellite data from Baja California, the equatorward distributional limit of the species on the coast of the East Pacific. As anticipated, giant kelp coverage showed high inter-annual variability modulated by the El Niño/La Niña Southern Oscillation (ENSO). The significant declines in 1991 to 1994, 1998, 2003, 2009 and 2016 were predicted when El Niño was strong or very strong (El Niño years 1991–1992, 1997–1998, 2002–2003; 2009–2010 and 2014–2016^76–78^). Conversely, during strong La Niña events, giant kelp recovered and achieved maximum coverage (e.g., La Niña years 1999–2000). The strong variation in kelp coverage in our predictions (i.e., the declines of 1991–1992 and 1997–1998 and recovery of 1999–2000) was also reported in additional studies^19,51^, with population changes occurring at large spatial scales (e.g., hundreds of km) and in orders of magnitude^19^, followed by fast recovery periods between 1 and 4 years^13^, as predicted.

Extreme climate conditions during El Niño years trigger marine heatwave events that have been linked to declines in giant kelp coverage, such as shown by our Mask R-CNN implementation^35,79,80^. Future climate conditions are projected to cause an increase in the frequency and intensity of marine heatwaves^81^, potentially causing permanent local extinctions for the species, with strong implications for ecosystem services^14^. In this line, the proposed method, aiming for automatic, regular, and updated monitoring of giant kelp forests and overcoming the need to perform repetitive tasks that can be time-consuming^42^, may be a key asset in facilitating well-informed biodiversity conservation, management and decision making (e.g., in the implementation of marine protected areas).

## Supplementary Information

Supplementary Information.

## Data Availability

The authors declare that all data used in modelling are openly available in Figshare at: https://doi.org/10.6084/m9.figshare.19935869. All code developed is permanently available at github.com/jorgeassis/maskRCNN.
