# Supplementary material for: Artificial intelligence convolutional neural networks map giant kelp forests from satellite imagery
Source: Sci Rep. 2022 Dec 23;12:22196. doi: 10.1038/s41598-022-26439-w (PMC9789120; doi:10.1038/s41598-022-26439-w)
Supplement: Supplementary file 1 — Supplementary Information. [file 41598_2022_26439_MOESM1_ESM.docx]

**Artificial intelligence convolutional neural networks map giant kelp forests from satellite imagery**

Marquez, L.^1^; Fragkopoulou, E.^1^; Cavanaugh, K.C.^2^; Houskeeper, H.F. ^2^; Assis, J^1^*.

**Supplementary Information**

Figures S1-S8: Overall losses per experiment assessed along the 50 epochs of training stages, and the epochs retrieving minimal losses (colored red).

Figures S9-S10: Example of Mask R-CNN predictions, including the bounding box detections of giant kelp.

Figure S1. Losses for experiment J01 with the testing dataset.

Figure S2. Losses for experiment J02 with the testing dataset.

Figure S3. Losses for experiment J03 with the testing dataset.

Figure S4. Losses for experiment J04 with the testing dataset.

Figure S5. Losses for experiment J05 with the testing dataset.

Figure S6. Losses for experiment J06 with the testing dataset.

Figure S7. Losses for experiment J07 with the testing dataset.

Figure S8. Losses for experiment J08. with the testing dataset.

Figure S9. Example of Mask R-CNN prediction, including the bounding box detections of giant kelp. Figure generated in R computing language ^1^ using an open-source Landsat satellite image, courtesy of the U.S. Geological Survey.

Figure S10. Example of Mask R-CNN prediction, including the bounding box detections of giant kelp. Figure generated in R computing language ^1^ using an open-source Landsat satellite image, courtesy of the U.S. Geological Survey.

**References**

1. R Development Core Team. *R: A Language and Environment for Statistical Computing*. *R: A Language and Environment for Statistical Computing* (R Foundation for Statistical Computing, 2021).
